# σ-Aromatic MAl_6_S_6_ (M = Ni, Pd, Pt) Stars Containing Planar Hexacoordinate Transition Metals

**DOI:** 10.3390/molecules28030942

**Published:** 2023-01-17

**Authors:** Li-Xia Bai, Jin-Chang Guo

**Affiliations:** Nanocluster Laboratory, Institute of Molecular Science, Shanxi University, Taiyuan 030006, China

**Keywords:** planar hexacoordinate metal (phM) clusters, global minimum, chemical bonding, 16-valence-electron counting, σ aromaticity

## Abstract

Hypercoordinate transition-metal species are mainly dominated by the 18-valence-electron (18ve) counting. Herein, we report ternary MAl_6_S_6_ (M = Ni, Pd, Pt) clusters with the planar hexacoordinate metal (phM) centers, which feature 16ve counting instead of the classic 18ve rule. These global-minimum clusters are established via unbiased global searches, followed by PBE0 and single-point CCSD(T) calculations. The phM MAl_6_ units are stabilized by six peripheral bridging S atoms in these star-like species. Chemical bonding analyses reveal that there are 10 delocalized electrons around the phM center, which can render the aromaticity according to the (4*n* + 2) Hückel rule. It is worth noting that adding an (or two) electron(s) to its π-type lowest unoccupied molecular orbital (LUMO) will make the system unstable.

## 1. Introduction

The design, synthesis, and characterization of new two-dimensional (2D) materials have attracted wide attention in recent years, for unexpected properties and wide applications [1,2,3,4,5]. As its units or fragments, the corresponding planar clusters have gradually become one of the focuses in cluster science. Planar hypercoordinate carbon species have been explored for half a century due to their exotic structures, unusual chemical bonding, as well as potential applications since Hoffmann et al. put forward the strategies for stabilizing planar tetracoordinate carbon (ptC) structures in 1970 [6,7,8,9,10,11,12,13]. A variety of ptC, planar pentacoordinate carbon (ppC), planar hexacoordinate carbon (phC) species were predicted theoretically or characterized experimentally [14,15,16,17,18,19,20,21,22,23,24,25]. The atypical planar hypercoordination is not limited to carbon, which can be extended to other main group elements and transition metals. Due to the electron-deficiency nature of boron, B*_n_*^−^ clusters in the size range of *n* = 3–40 have been confirmed to assume planar or quasi-planar structures, in which some species can possess even higher coordination numbers [26,27]. Planar hepta- and octacoordinate boron (p7B, p8B) centers can be stabilized in the beautiful molecular wheels B_8_^−^ and B_9_^−^, which were experimentally observed by Zhai in 2003 [28]. The wheel-like ppB B_6_H_5_^+^, ppBe BeCu_5_, p8Be BeB_8_^2−^, p7Sc ScCu_7_, ppAl Cu_5_Al^2+^, ppGa Cu_5_Ga^2+^, and star-like ppB BBe_5_Au_5_, phGa GaBe_6_Au_6_ were identified as the global minima [29,30,31,32,33,34,35]. Using size-selected anion photoelectron spectroscopy combined with ab initio calculations, a series of transition-metal-centered monocyclic boron wheel clusters M©B*_n_* (*n* = 8–10) were reported by Wang and Boldyrev [36]. Among them, *D*_8*h*_-Co©B_8_^−^, *D*_9*h*_-Ru©B_9_^−^, *D*_10*h*_-Ta©B_10_^−^ are the most representative molecular wheels, containing the planar octacoordinate cobalt, planar nonacoordinate ruthenium, and planar decacoordinate tantalum, respectively [37,38]. More importantly, an effective principle was proposed for designing the stable M©B*_n_*^k−^, that is *x* =12-*n*-*k*, where *x* is the required valence of the transition-metal M atom. Obviously, Co©B_8_^−^ and Ru©B_9_^−^ follow the formula exactly. For *D*_10*h*_-Ta©B_10_^−^, the corresponding electron counting formula needs to upgrade to *x* =16-*n*-*k*. It should be noted that these beautiful molecular wheels possess σ and π double aromaticity. In 2015, a series of planar wheel-type *D*_6*h*_ M©B_6_H_6_^−/0/+^ (M = Mn, Fe, and Co) clusters were theoretically designed by Hou [39]. The H-bridged Be*_n_*H*_n_* rings can be used to stabilize the ppC, phB, and p7Au [40,41,42]. However, it is a failure to stabilize the transition metal atom with a Be*_n_*H*_n_* ring. Recently, the ppB BAl_5_S_5_^+^, ppC CAl_5_O_5_^+^, and CB_5_S_5_^+^ molecular stars were predicted in theory [43,44,45]. Very recently, the MB_7_O_7_^+^ (M = Ni, Pd, Pt) stars were reported as the global minima by Wu, which contain the p7M centers and possess the σ aromaticity alone [46]. To better broaden the research field of the planar hypercoordinate transition metals species, it is highly desirable to design the phM clusters, which is the theme of our present study. To host a planar hypercoordinate transition metal center, the M atom and surrounding ligands ring must match both geometrically and electronically. One interesting question arises regarding how to design molecular stars containing the planar hexacoordinate Ni/Pd/Pt centers.

**Scheme 1:**
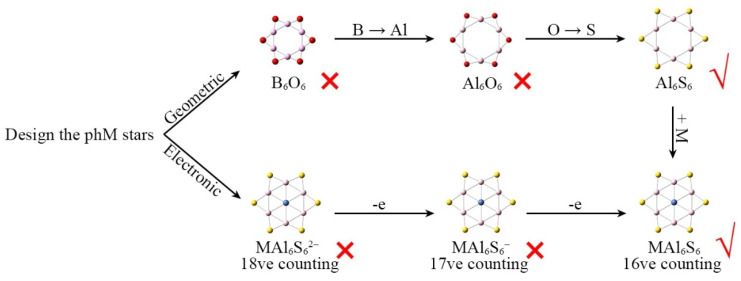
Basic idea of designing phM MAl_6_S_6_ (M = Ni, Pd, Pt) clusters.

Our design idea is shown in Scheme 1. Geometrically, the star-like B_6_O_6_ ring is too small to hold a Ni atom. It is imperative to increase the geometrical size of the ligand ring. Our preliminary calculations show that *D*_6*h*_ NiAl_6_O_6_ is only a transition state structure. Can we stabilize the Ni/Pd/Pt atoms using a larger Al_6_S_6_ ring? The answer is positive. Geometrically, the Al_6_S_6_ ring is suitable to hold the Ni/Pd/Pt transition metal center. As we all know, 18-valence-electron (18ve) counting is popular for transition metal species. It is easy to think of MAl_6_S_6_^2−^ (M = Ni, Pd, Pt) systems first. However, MAl_6_S_6_^2−^ (M = Ni, Pd, Pt) are the third-order saddle points on the corresponding energy surfaces, at the PBE0/def2-TZVP level. Removing one electron from MAl_6_S_6_^2−^, the MAl_6_S_6_^−^ (M = Ni, Pd, Pt) are formed. Unfortunately, MAl_6_S_6_^−^ (M = Ni, Pd, Pt) are still unstable, which are only the second-order saddle points. Thus, both 18ve and 17ve counting are invalid. Frequency analyses indicate that neutral MAl_6_S_6_ (M = Ni, Pd, Pt) are the true minima at the PBE0/def2-TZVP level. Thus, 16ve counting seems to be an appropriate electronic rule to design the phM species.

The present paper reports ternary S-bridged MAl_6_S_6_ (M = Ni, Pd, Pt) molecular stars, which contain the planar hexacoordinate transition metal centers. Interestingly, MAl_6_S_6_ (M = Ni, Pd, Pt) possesses 10σ aromaticity alone, whose transition metal centers follow the 16ve counting instead of the typical 18ve rule. The MAl_6_S_6_ (M = Ni, Pd, Pt) species reported in this paper will provide new candidates for further exploration of 2D materials.

## 2. Computational Details

The potential energy surfaces of MAl_6_S_6_ (M = Ni, Pd, Pt) were explored by the Coalescence Kick (CK) algorithm [47,48], at the PBE0/Lanl2DZ level of theory [49]. Both singlet and triplet surfaces were considered. More than 8000 stationary points (4000 singlets and 4000 triplets) were probed for each of the MAl_6_S_6_ (M = Ni, Pd, Pt) clusters. Subsequent structural optimizations were carried out for the low-lying isomers using the PBE0 method with the def2-TZVP basis set [50]. Frequency calculations were performed at the same level to ensure that all the structures are true minima on the potential energy surface. The energies were refined by the single point CCSD(T)/def2-TZVP calculations at the PBE1PBE/def2-TZVP geometries [51]. The final relative energies were determined by the CCSD(T)/def2-TZVP energy plus the PBE0/def2-TZVP zero-point energy corrections. Born–Oppenheimer molecular dynamic (BOMD) simulations were performed at the PBE0/def2-SVP level [52].

To obtain Wiberg bond indices (WBIs) and natural atomic charges, natural bond orbital (NBO) analyses were conducted at the PBE0/def2-TZVP level using NBO6.0 [53]. The atomic composition of canonical molecular orbitals (CMOs), adaptive natural density partitioning (AdNDP), and electron localization function (ELF) analyses were accomplished using the Multiwfn program [54,55,56]. Nucleus-independent chemical shifts (NICSs) and iso-chemical shielding surfaces in the z direction (ICSS_zz_) were calculated to assess aromaticity [57,58]. All electronic structure calculations were carried out using the Gaussian 16 package [59]. Molecular structures, CMO pictures, AdNDP, and ELF results were visualized using CYLview [60] and GaussView, respectively.

## 3. Results and Discussion

### 3.1. Structures and Stability

**Figure 1:**
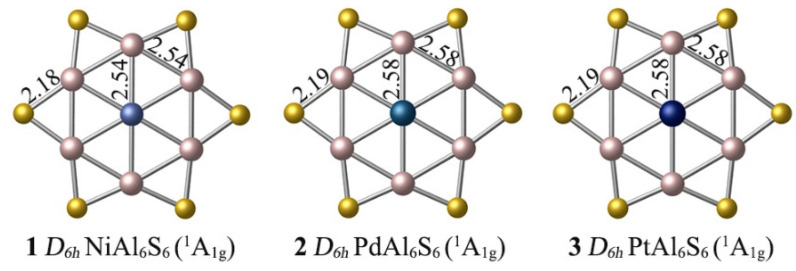
Optimized global-minimum structures **1**–**3** of MAl_6_S_6_ (M = Ni, Pd, Pt) at the PBE0/def2-TZVP level. The bond distances are labeled in angstroms.

**Figure 2:**
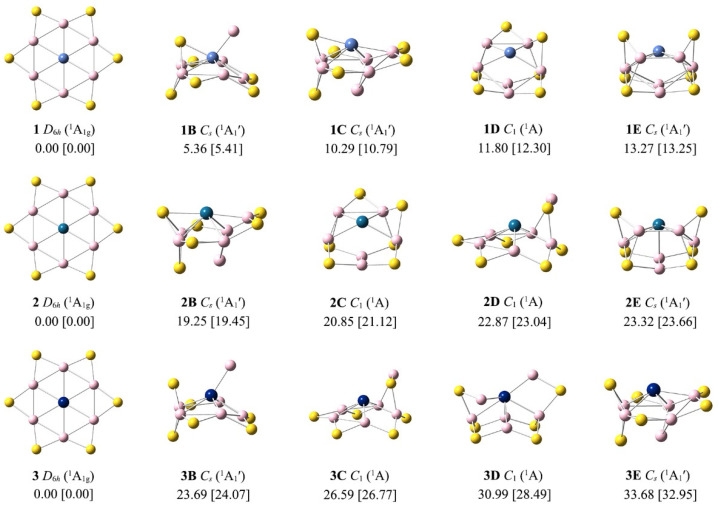
Optimized GM structures **1**–**3** of the MAl_6_S_6_ (M = Ni, Pd, Pt) clusters at the PBE0/def2-TZVP level, along with their four lowest-lying isomers (***n*B**–***n*E**). Relative energies are listed in kcal mol^−1^ at the single-point CCSD(T) level using their PBE0 geometries, with zero-point energy (ZPE) corrections at PBE0. Shown in square brackets for comparison are the energetics at single-point CCSD(T)/def2-TZVP//B3LYP/def2-TZVP level, including ZPE corrections at B3LYP.

The optimized global minima (GMs) structures of ternary MAl_6_S_6_ (M = Ni, Pd, Pt) clusters were depicted in Figure 1, at the PBE0/def2-TZVP level. The phM GMs (**1**–**3**) assume the perfect *D*_6*h*_ symmetry, which is composed of the M center, Al_6_ ring, and six S bridges at the periphery. Geometrically, the star-like Al_6_S_6_ ring is suitable for hosting these hexacoordinate planar transition metals (phMs) with the high symmetry of *D*_6*h*_, which has good tolerance and can be adjusted according to the size of the central atom. Frequency analyses indicate that the empty *D*_6*h*_ Al_6_S_6_ ring is unstable, which is only a transition state at PBE0/def2-TZVP level. Introducing a transition metal atom into its center can help to stabilize it. Alternative optimized low-lying structures are shown in Figure 2. To check for consistency in terms of structures and energetics, the corresponding B3LYP/def2-TZVP calculations and vibrational analyses were further done for **1**–**3** and the low-lying isomers, as well as the single-point CCSD(T)/def2-TZVP//B3LYP/def2-TZVP calculations [61,62]. As shown in Figure 2, the CCSD(T)//B3LYP energetics data (in square brackets) are closely consistent with those at single-point CCSD(T)//PBE0. Thus, we shall only discuss the PBE0 and CCSD(T)//PBE0 data in the following discussion.

At the CCSD(T)//PBE0 level, the GMs **1**, **2**, **3** lie 5.36, 19.25, and 23.69 kcal mol^−1^ lower than the second low-lying isomers **1B**, **2B**, and **3B**, respectively. Their optimized Cartesian coordinates for GMs **1**–**3** and the low-lying isomers **1B**–**3E** are provided in Table S1 (ESI†). In these low-lying isomers, all Al atoms tend to coordinate directly to the M center, whereas most of the S atoms are situated on the periphery and link with Al atoms as the bridges (μ^2^-S). It should be noted that there is one μ^3^-S atom is bound to the M center as the face-capping group in **1B**/**1C**/**1D**/**2B**/**2D**/**3B**/**3C**/**3E**. However, there is no terminal μ^1^-S atom in these low-lying isomers. The central M atom has a coordination number of six/seven, while the coordination number of the Al atom is three/four in **1B**–**3E**. The most stable triplet structures are obviously higher than **1E**, **2E**, and **3E** in energy at the CCSD(T) level, respectively. Thus, the phM **1**, **2**, and **3** stars are the GMs on the potential energy surfaces.

The bond distances, Wiberg bond indices (WBIs), and natural population analysis (NPA) charges can help us to explore the bonding characters of **1**–**3**, which are shown in Figure 1 and Table 1. As shown in Figure 1, the calculated Ni–Al bond distance in **1** is 2.54 Å, which is longer than the Ni–Al covalent single bond length (2.36 Å) based on covalent atomic radii proposed by Pyykkö [63]. Because of the perfect *D*_6*h*_ symmetry, the Al–Al bond distance in **1** is also 2.54 Å, which is close to the recommended single bond (2.52 Å). The Al–S distance 2.18 Å, is between the Al-S single bond (2.29 Å) and Al=S double bonds (2.07 Å). Considering the differences in electronegativity of the elements (Ni:1.9, Al:1.5, S:2.5), Ni-Al and Al-S bonding can be sort of polar in nature. Thus, it is not easy to judge the bond strength just by the bond distances. WBIs and NPA charges offer valuable bonding information. As shown in Table 1, the WBI_Ni–Al_ 0.22 and WBI_Al–Al_ 0.40, are less than 0.5, indicating their delocalized bonding nature. The WBI_Al–S_ in **1** is 0.88, suggesting that the Al–S bonding is quite strong, which is consistent with the conclusions from the analyses of bond distances. With the increases in the size of central M atoms, the changes in the corresponding bond distances have almost the same trends. Interestingly, the Pd–Al in **2** has exactly equal bond distance with the Pt–Al (2.58 Å) in **3**. The WBI_Pd–Al_ and WBI_Pt–Al_ are 0.23 and 0.25, respectively. It should be noted that the periphery Al–S bond distances in **1**–**3** seem to be almost unchanged (from 2.18 to 2.19 Å), as well as their WBIs. Indeed, the bridging S atoms can help to make the relatively flexible Al_6_ ring more rigid to some extent.

Due to the electronegativity differences, there is obvious electron transfer from the Al atoms to the central M and periphery S atoms. As shown in Table 1, the NPA charges on Ni, Al, and S atoms are −0.30, +0.84, and −0.79 |e|, respectively, thus **1** features the negative–positive–negative charge distribution pattern, which favors the stabilization of the species via Coulomb attractions. Since Pd, Pt and Ni are in the same group in the periodic table, the charge distributions in **2** and **3** are basically similar to those of **1**. It should be noted that the transition metals carry only a small number of negative charges, making them easier to stabilize. According to NPA charge data, MAl_6_S_6_ (M = Ni, Pd, Pt) can approximately be formulated as [MAl_6_]^6+^[S_6_]^6−^.

**Figure 3:**
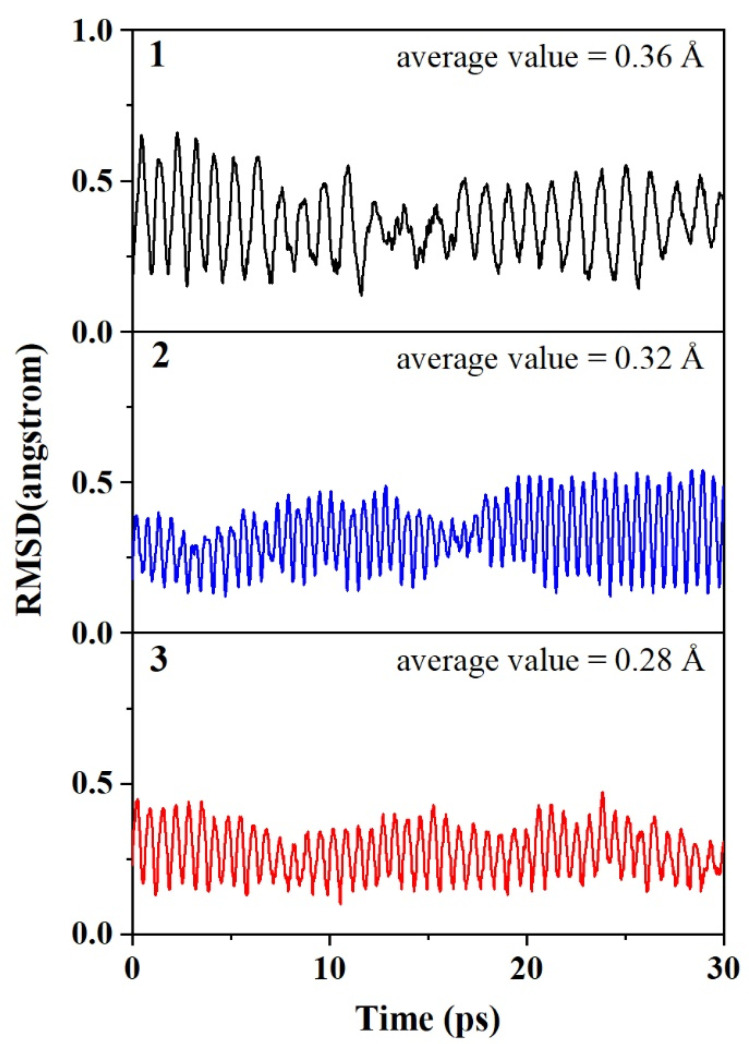
Calculated root-mean-square deviations (RMSDs) of GM clusters **1**–**3** during the Born-Oppenheimer molecular dynamics (BOMD) simulations at 298 K.

For a thermodynamically stable cluster, its kinetic stability is equally important for experimental realization. Are these perfect phM MAl_6_S_6_ (M = Ni, Pd, Pt) stars dynamically stable? Born–Oppenheimer molecular dynamics (BOMD) simulations were performed for clusters **1**–**3**, at the PBE0/def2-SVP level, for 30 ps at room temperature (298 K). The root-mean-square deviations (RMSD, relative to the PBE0/def2-SVP optimized structures) of the structures are depicted in Figure 3. The average RMSD values of **1**–**3** are in the 0.28~0.36 Å range, indicating that their original structures can be maintained during the 30 ps simulations. As a technical note, the major spikes in **1** are due to the inversion vibration of Al/S up and down the molecular planes, suggesting that the Al_6_ ring is relatively soft. As the size of the central M atom increases, the vibration decreases. The BOMD data suggest that **1**–**3** species also have reasonable kinetic stability against isomerization and decomposition.

### 3.2. Chemical Bonding

**Figure 4:**
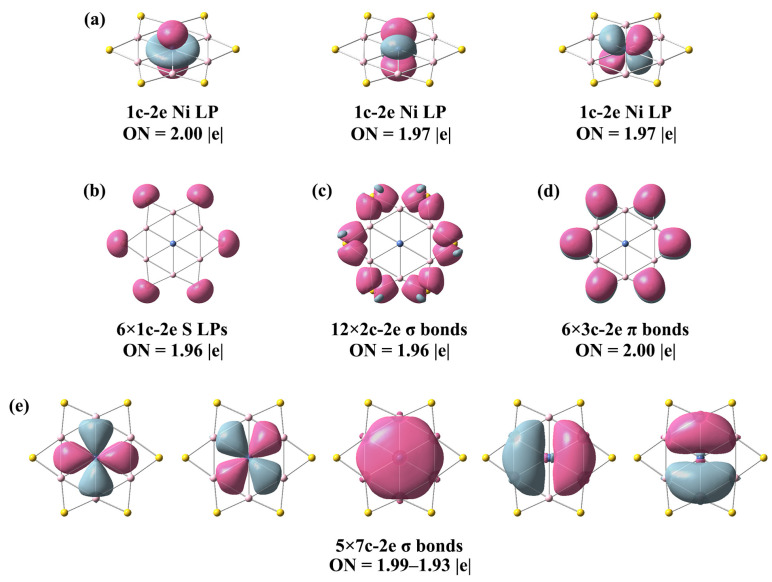
The AdNDP bonding pattern of **1**. Occupation numbers (ONs) are shown.

To further understand the electronic structures and stability of MAl_6_S_6_ (M = Ni, Pd, Pt) clusters, it is essential to perform in-depth chemical bonding analyses. Here, we chose to carry out AdNDP analyses for these phM stars. AdNDP is an extension of the NBO analysis, which recovers not only the Lewis bonding elements (lone pairs and 2c-2e bonds) but also delocalized *n*c-2e bonds. The AdNDP scheme for NiAl_6_S_6_ is illustrated in Figure 4, which is relatively straightforward for comprehension. There are 64 valence electrons in NiAl_6_S_6_, which can be attributable to five subsets from (a) to (e), according to the CMO orbital composition and structural characteristics. As shown in Figure 4a, there are three 1c-2e lone pairs (LPs) on the central Ni atom, with the occupation numbers (ON) from 2.00 to 1.97 |e|. Subset (b) indicates that there are six 1c-2e LPs of the periphery S atoms (ON = 1.96 |e|). Subset (c) contains 12 typical 2c-2e Al-S localized σ bonds on the peripheral Al_6_S_6_ ring (ON = 1.96 |e|). In Subset (d), there are six delocalized 3c-2e π bonds on the Al-S-Al triangles, with the ON 2.00 |e|. The residual 10 electrons can correspond to five 7c-2e delocalized σ bonds on the NiAl_6_ unit, which are depictured in subset (e). Thus, the phM NiAl_6_S_6_ possesses 10σ aromaticity, according to the classical (4*n* + 2) Hückel rule. The orbital composition of occupied CMOs for **1** is listed in Table S2, which further supports the above AdNDP results. The AdNDP bonding patterns of **2** and **3** are very similar to **1**, which are shown in Figure S1 and Figure S2, respectively.

**Figure 5:**
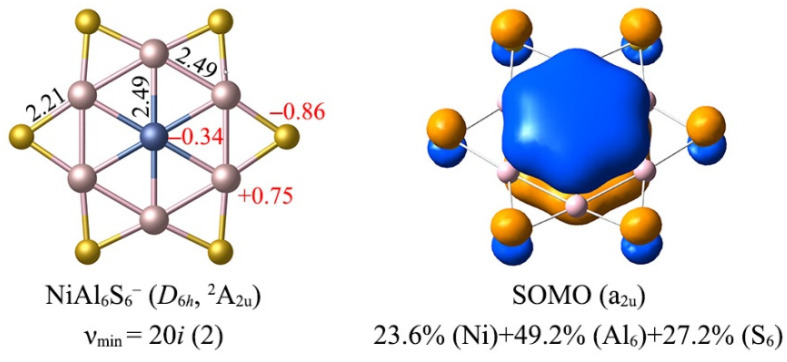
Optimized structures *D*_6*h*_ NiAl_6_S_6_^−^ at the PBE0/def2-TZVP level. The bond distances (in Å; black color), atomic nature charges ((in |e|; red color), the lowest vibrational frequency ν_min_ (cm^−1^) and the number of imaginary frequencies are shown. The SOMO and its compositions are also listed.

A total of 64 valence electrons are ideal for these σ aromatic phM stars, and even one additional electron can deteriorate their electronic stability. Table S3 listed the LUMOs and atomic composition of **1**–**3**. For such delocalized orbitals, intuitively, adding electrons to them will be beneficial to the stability of the systems. However, the result is counter-intuitive. As depictured in Figure 5, *D*_6*h*_ NiAl_6_S_6_^−^ (^2^A_2u_) is only a second-order saddle point on the potential energy surface, with two small imaginary frequencies at the PBE0/def2-TZVP level. It should be noted that the singly occupied molecular orbital (SOMO) of NiAl_6_S_6_^−^ is a typical delocalized π orbital. In general, the electron on π-SOMO should be beneficial to the stability of the system. Even then, the *D*_6*h*_ NiAl_6_S_6_^−^ anion is not stable. Why is the phM NiAl_6_S_6_^−^ star unstable? We briefly answer this question. The electron on SOMO has both positive and negative effects on the stability of the plane structure of NiAl_6_S_6_^−^. On the one hand, π delocalization can help to disperse electrons from the central transition metal Ni atom. However, the charge on Ni is only −0.34 |e|, which makes this effect contribute a little to the stability of the NiAl_6_S_6_^−^ star. In addition, although the SOMO is a bonding orbital in general, it has some antibonding components. The π-LPs of S atoms play a crucial role in the anti-bonding characters, which account for 27.2% of the total. The combination of these two factors makes the electrons on SOMO contribute very little to the stability of the system. On the other hand, both Ni-Al and Al-Al bond distances are equal to 2.49 Å, which is obviously shorter than those in NiAl_6_S_6_ (2.54 Å). According to the NPA distribution, the charge of the Al atom is +0.75 |e| in NiAl_6_S_6_^−^. The relatively short Al-Al bond distances make the electrostatic repulsion between them increase obviously, which makes the plane structure of *D*_6*h*_ NiAl_6_S_6_^−^ become unstable. Similarly, *D*_6*h*_ NiAl_6_S_6_^2−^ dianion is the third-order saddle point on the potential energy surface, whose Ni center satisfies the 18ve rule. Thus, 18ve counting is not a prerequisite for the planar hypercoordinate transition metals species.

### 3.3. σ-Aromaticity

**Figure 6:**
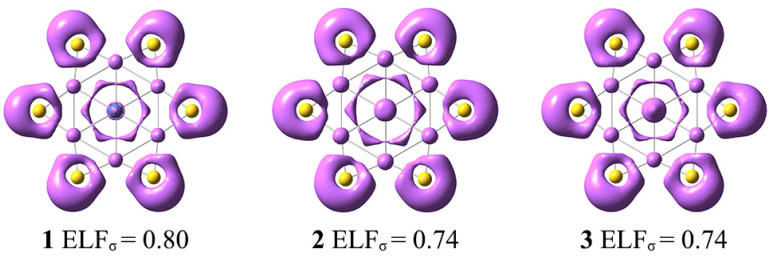
Electron localization function (ELF) of σ electron clouds for **1**–**3**. The approximated bifurcation values are shown.

In general, many beautiful planar molecules are associated with aromaticity, which is very important for the stability of the system. Aromaticity can be described not only qualitatively but also quantitatively. To further strengthen the AdNDP analyses presented above, detailed ELF analyses were performed for the MAl_6_S_6_ (M = Ni, Pd, Pt) stars. As shown in Figure 6, the bifurcation values of ELF_σ_ in **1**–**3** are 0.80, 0.74, and 0.74, respectively. Thus, the MAl_6_S_6_ (M = Ni, Pd, Pt) stars are σ aromatic according to the ELF criteria, which are consistent with our conclusion of AdNDP analyses.

**Figure 7:**
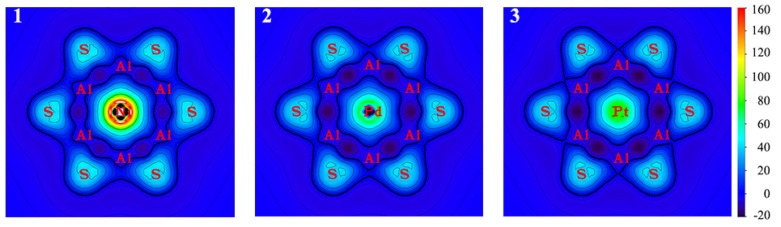
Color-filled maps of ICSS_zz_ (in ppm) at the molecular plane for the **1**–**3** clusters. Positive values indicate aromaticity.

As an independent, semi-quantitative measure of aromaticity in the systems, NICS plays an important role in the characterization of aromaticity. The negative NICS(0) and NICS(1) values can reflect σ and π aromaticity, respectively. As shown in Figure S3, the NICS(0) values at the Al-M-Al triangles are in the range of −32.0~−63.8 ppm for **1**–**3**, suggesting they possess typical σ aromaticity. For the Al-S-Al triangles of **1**–**3**, the NICS(1) values are from −4.0 to −6.5 ppm, indicating they have a certain degree of π aromaticity. However, evaluating NICS values at some point seems to be inadequate. To more intuitively observe the aromaticity, the color-filled maps of ICSS_zz_(0) of **1**–**3** are plotted in Figure 7. It should be noted that positive ICSS_zz_ values indicate diatropic ring currents and aromaticity. The calculated ICSS_zz_ data provide a quantitative assessment, fully supporting the idea of σ aromaticity in MAl_6_S_6_ (M = Ni, Pd, Pt) stars.

### 3.4. Simulated IR Spectrums

**Figure 8:**
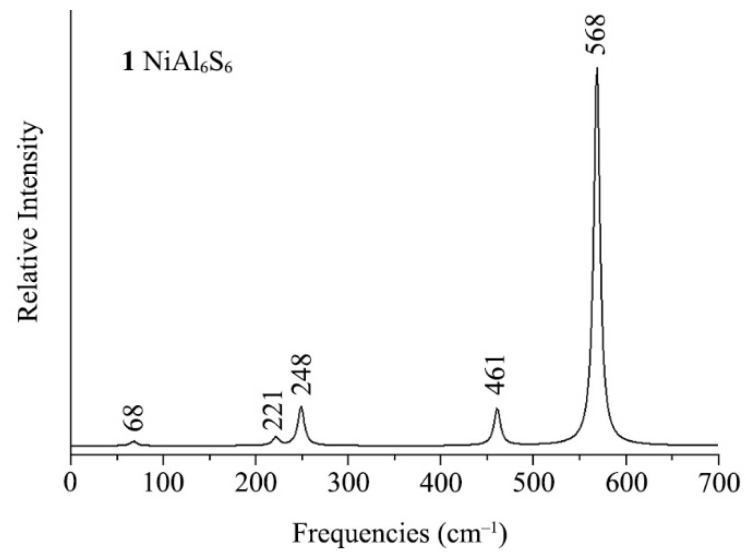
Calculated IR spectrum of NiAl_6_S_6_ at the PBE0/def2-TZVP level.

To facilitate future experimental characterization, the IR spectrums of the phM stars **1**, **2**, and **3** were simulated at the PBE0/def2-TZVP level. As depictured in Figure 8, the strongest IR absorption peak occurs at 568 cm^−1^, which mainly originates from inplane asymmetrical Al-S stretching vibrations. The peak at 461 cm^−1^, is mainly generated by coupled vibrations of Al-S, Al-Ni, and Al-Al in-plane asymmetrical stretching vibrations. The weak peak at 248 cm^−1^, corresponds to asymmetrical Ni-Al in-plane stretching vibrations. The weak peak at 67 cm^−1^ is the result of the up-and-down movements of the phNi center within the Al_6_ ring along the molecular axis. As shown in Figure S4, the locations and intensity of absorption peaks in **2** (PdAl_6_S_6_) and **3** (PtAl_6_S_6_) are basically like those of **1**.

## 4. Summary

In summary, we have designed the phM MAl_6_S_6_ (M = Ni, Pd, Pt) stars. Based on extensive searches and high-level calculations, these perfect phM stars turned out to be the global minima on the potential energy surfaces. In addition, the dynamic simulations revealed that they possess good kinetic stabilities. The formation of peripheral 2c-2e Al-S σ bonds, 3c-2e Al-S-Al π bonds, and the 10σ aromaticity contribute to the stabilization of phM-containing structures in MAl_6_S_6_ (M = Ni, Pd, Pt) species. Thus, these phM species may be targeted in future experiments and enrich the planar hypercoordinate transition metals chemistry.

## Figures and Tables

**Table 1:** The lowest vibrational frequencies (ν_min_/cm^−1^), HOMO-LUMO gaps (Gap/eV), Wiberg bond indices, and atomic nature charges (q, |e|) of MAl_6_S_6_ (M = Ni, Pd, Pt) at the PBE0/def2-TZVP level.

Species	*ν*_min_	Gap	WBI_M-Al_	WBI_Al-Al_	WBI_Al-S_	q_M_	q_Al_	q_S_
**1** NiAl_6_S_6_	17	3.24	0.22	0.40	0.88	−0.30	0.84	−0.79
**2** PdAl_6_S_6_	27	3.23	0.23	0.38	0.87	−0.42	0.87	−0.80
**3** PtAl_6_S_6_	24	3.26	0.25	0.36	0.87	−0.54	0.89	−0.80

## Data Availability

Not applicable.

## Supplementary Materials

The following supporting information can be downloaded at: https://www.mdpi.com/article/10.3390/molecules28030942/s1, Table S1: Cartesian coordinates for global-minimum (GM) clusters 1–3 of the MAl6S6 (M = Ni, Pd, Pt) series at the PBE0/def2-TZVP level, along with four lowest-lying nB–nE isomeric structures.; Table S2: Orbital composition analysis for the canonical molecular orbitals (CMOs) of GM NiAl6S6 (1, D6h, 1A1g) cluster. Main components greater than 15% are shown in bold; Table S3: Orbital composition analysis for the lowest unoccupied molecular orbitals (LUMOs) of the MAl6S6 (M = Ni, Pd, Pt) series; Figure S1: The AdNDP bonding pattern of PdAl6S6. Occupation numbers (ONs) are shown; Figure S2: The AdNDP bonding pattern of PtAl6S6. Occupation numbers (ONs) are shown; Figure S3: Nucleus independent chemical shifts (NICSs) for clusters 1–3. Here the NICS(0) data (blue color) are calculated at the center of the Al-M-Al (M = Ni, Pd, Pt) triangle, whereas the NICS(1) values (red color) are at 1 Å above the center of the Al-S-Al triangle; Figure S4: Calculated IR spectrums of PdAl6S6 and PtAl6S6 at the PBE0/def2-TZVP level.
