# Supplementary material for: σ-Aromatic MAl6S6 (M = Ni, Pd, Pt) Stars Containing Planar Hexacoordinate Transition Metals
Source: Molecules. 2023 Jan 17;28(3):942. doi: 10.3390/molecules28030942 (PMC9920543; doi:10.3390/molecules28030942)

Supplementary Material

σ-Aromatic MAl_6S_6 (M = Ni, Pd, Pt) Stars Containing Planar Hexacoordinate Transition Metals

Li-Xia Bai and Jin-Chang Guo *

Nanocluster Laboratory, Institute of Molecular Science, Shanxi University, Taiyuan 030006, China; bailixia2021@163.com
* Correspondence: guojc@sxu.edu.cn

Table S1. Cartesian coordinates for global-minimum (GM) clusters **1**–**3** of the MAl_6S_6 (M = Ni, Pd, Pt) series at the PBE0/def2-TZVP level, along with four lowest-lying ***n*B**–***n*E** isomeric structures.

Table S2. Orbital composition analysis for the canonical molecular orbitals (CMOs) of GM $NiAl_6S_6$ (**1**, D_{6h}, $^1A_{1g}$) cluster. Main components greater than 15% are shown in bold.

Table S3. Orbital composition analysis for the lowest unoccupied molecular orbitals (LUMOs) of the MAl_6S_6 (M = Ni, Pd, Pt) series.

Figure S1. The AdNDP bonding pattern of $PdAl_6S_6$. Occupation numbers (ONs) are shown.

Figure S2. The AdNDP bonding pattern of $PtAl_6S_6$. Occupation numbers (ONs) are shown.

Figure S3. Nucleus independent chemical shifts (NICSs) for clusters **1**–**3**. Here the NICS(0) data (blue color) are calculated at the center of the Al-M-Al (M = Ni, Pd, Pt) triangle, whereas the NICS(1) values (red color) are at 1 Å above the center of the Al-S-Al triangle.

Figure S4. Calculated IR spectrums of $PdAl_6S_6$ and $PtAl_6S_6$ at the PBE0/def2-TZVP level.

Table S1. Cartesian coordinates for global-minimum (GM) clusters **1–3** of the MAl_6S_6 (M = Ni, Pd, Pt) series at the PBE0/def2-TZVP level, along with four lowest-lying ***n*B–*n*E** isomeric structures.

1 $NiAl_6S_6$ (D_{6h}, $^1A_{1g}$)

Ni	0.00000000	0.00000000	0.00000000
Al	0.00000000	2.53498700	0.00000000
Al	−2.19536300	1.26749400	0.00000000
Al	2.19536300	1.26749300	0.00000000
Al	2.19536300	−1.26749400	0.00000000
Al	0.00000000	−2.53498700	0.00000000
Al	−2.19536300	−1.26749300	0.00000000
S	−1.98667600	3.44102400	0.00000000
S	1.98667600	3.44102400	0.00000000
S	3.97335200	0.00000000	0.00000000
S	1.98667600	−3.44102400	0.00000000
S	−1.98667600	−3.44102400	0.00000000
S	−3.97335200	0.00000000	0.00000000

1B $NiAl_6S_6$ (C_s, $^1A'$)

Ni	0.67460200	0.19444800	0.00000000
Al	−0.92387700	1.95041800	0.00000000
Al	0.19351400	−1.83175300	1.38505000
Al	0.19351400	−1.83175300	−1.38505000
Al	2.17457400	1.74292300	0.00000000
Al	−0.08436800	0.74303800	−2.18111900
Al	−0.08436800	0.74303800	2.18111900
S	−0.08436800	−1.07057000	−3.43346600
S	−1.45421900	2.48469800	−2.06270100
S	−1.27640800	−2.71849400	0.00000000
S	−0.08436800	−1.07057000	3.43346600
S	1.97947600	−1.68172500	0.00000000
S	−1.45421900	2.48469800	2.06270100

1C $NiAl_6S_6$ (C_s, $^1A'$)

Ni	0.89493600	0.18857400	0.00000000
Al	−1.69017400	−0.25659500	0.00000000
Al	−0.52794500	0.71487400	2.11094400
Al	0.36169200	−1.72833600	−1.40043700
Al	0.36169200	−1.72833600	1.40043700
Al	−0.52794500	0.71487400	−2.11094400
Al	−0.21701600	2.30513000	0.00000000
S	−1.19350700	−2.64301300	0.00000000
S	2.12988900	−1.73767000	0.00000000
S	0.18656100	−0.92324400	−3.43384400
S	0.18656100	−0.92324400	3.43384400
S	−0.52794500	2.93980400	2.07949800
S	−0.52794500	2.93980400	−2.07949800

1D $NiAl_6S_6$ (C_1, ^1A)

Ni	−0.00994900	−0.10477900	−1.05421000
Al	0.14649000	0.49894800	1.53118700
Al	2.34013800	0.08478700	0.46138700
Al	−1.96328800	−0.99705000	−0.02846100
Al	0.55646900	−2.00192000	0.28476100
Al	−1.62404900	1.64300600	−0.62907800
Al	0.78533700	2.07657300	−0.68678300
S	−0.58216500	2.61788400	1.23284000
S	−0.99288300	−1.49985400	1.98127500
S	−0.81414100	−2.29792500	−1.45734700
S	−3.50877700	0.51089500	−0.29614800
S	2.73827700	−2.10026200	0.28873600
S	2.98120600	1.89284700	−0.66256200

1E $NiAl_6S_6$ (C_s, $^1A'$)

Ni	−0.92651300	−0.24786300	0.00000000
Al	0.08428900	−2.07002200	1.23503700
Al	−0.09184000	0.46054300	2.10353400
Al	0.45194600	2.16173400	0.00000000
Al	1.67946500	0.03531900	0.00000000
Al	0.08428900	−2.07002200	−1.23503700

2 $PdAl_6S_6$ (D_{6h}, $^1A_{1g}$)

Pd	0.00000000	0.00000000	0.00000000
Al	0.00000000	2.58196200	0.00000000
Al	2.23604500	1.29098100	0.00000000
Al	−2.23604500	1.29098100	0.00000000
Al	−2.23604500	−1.29098100	0.00000000
Al	0.00000000	−2.58196200	0.00000000

Al	−0.09184000	0.46054300	−2.10353400
S	−1.68829300	−1.09728900	−2.20871600
S	−0.22309800	2.64641500	2.06595000
S	1.86233900	−0.91709700	2.11807400
S	−1.68829300	−1.09728900	2.20871600
S	1.86233900	−0.91709700	−2.11807400
S	−0.22309800	2.64641500	−2.06595000

Al	2.23604500	−1.29098100	0.00000000
S	2.00134900	3.46643800	0.00000000
S	−2.00134900	3.46643800	0.00000000
S	−4.00269800	0.00000000	0.00000000
S	−2.00134900	−3.46643800	0.00000000
S	2.00134900	−3.46643800	0.00000000
S	4.00269800	0.00000000	0.00000000

2B $PdAl_6S_6$ (C_s, $^1A'$)

Pd	0.94425900	0.29292700	0.00000000
Al	−1.89134100	−0.33946000	0.00000000
Al	−0.77327300	0.64643800	2.08625900
Al	0.40467500	−1.75743300	−1.37851000
Al	0.40467500	−1.75743300	1.37851000
Al	−0.77327300	0.64643800	−2.08625900
Al	−0.42487200	2.32881200	0.00000000
S	−1.19361200	−2.63791400	0.00000000
S	2.17431600	−1.96983400	0.00000000
S	0.16599700	−0.91092200	−3.38009600
S	0.16599700	−0.91092200	3.38009600
S	−0.77327300	2.88822400	2.08278300
S	−0.77327300	2.88822400	−2.08278300

2C $PdAl_6S_6$ (C_1, ^1A)

Pd	−0.03358200	0.10282800	−1.08965500
Al	−0.31475700	−0.52137200	1.73044500
Al	−2.30136900	0.28775900	0.50066100
Al	2.11229200	0.65629500	0.08203800
Al	−0.14796000	2.09015400	0.41540200
Al	1.30484100	−1.96252700	−0.57971600
Al	−1.16240600	−2.00996500	−0.61155100
S	0.11770000	−2.69820700	1.30909800
S	1.20693800	1.18917600	2.12551100
S	1.43598100	2.41372400	−1.12871100
S	3.33212200	−1.11977900	−0.24766000
S	−2.29376800	2.51774300	0.37429800
S	−3.28857100	−1.41231800	−0.54881800

2D $PdAl_6S_6$ (C_1, ^1A)

Pd	0.04861600	0.00524400	0.75912000
Al	−1.74586700	1.23903600	−0.57162600
Al	1.18526900	−2.07944400	−0.03216200
Al	2.48059400	0.24719200	0.23668100
Al	−2.74840500	−0.89444100	1.58343000
Al	0.85384200	2.16953100	−0.20054400
Al	−1.32532000	−1.76466800	−0.45058900
S	3.05543600	2.26875700	−0.37449400
S	−0.76014500	3.02858800	−1.39283600
S	−0.08485900	−3.46254900	−1.14923600
S	−2.72091500	−0.44009400	−1.60299100
S	3.35175000	−1.75923600	0.20237600
S	−1.92488200	1.22923100	1.67549400

2E $PdAl_6S_6$ (C_s, $^1A'$)

Pd	0.11113000	−0.97125700	0.00000000
Al	2.03642500	0.09288700	1.25833800
Al	−0.44578900	−0.00343500	2.20900500
Al	−2.16302600	0.44501300	0.00000000
Al	−0.15522200	1.85543300	0.00000000
Al	2.03642500	0.09288700	−1.25833800
Al	−0.44578900	−0.00343500	−2.20900500
S	1.21763500	−1.41318300	−2.70093500
S	−2.63601300	−0.16722000	2.10009500
S	0.90802500	1.96934900	2.02544900
S	1.21763500	−1.41318300	2.70093500
S	0.90802500	1.96934900	−2.02544900
S	−2.63601300	−0.16722000	−2.10009500

3 $PtAl_6S_6$ (D_{6h}, $^1A_{1g}$)

Pt	0.00000000	0.00000000	0.00000000
Al	0.00000000	2.57597200	0.00000000
Al	2.23085700	1.28798600	0.00000000
Al	−2.23085700	1.28798600	0.00000000
Al	−2.23085700	−1.28798600	0.00000000
Al	0.00000000	−2.57597200	0.00000000

3B $PtAl_6S_6$ (C_s, $^1A'$)

Pt	0.62517100	0.25529800	0.00000000
Al	−1.10890500	1.95446800	0.00000000
Al	0.06991800	−1.91387500	1.39161300
Al	0.06991800	−1.91387500	−1.39161300
Al	2.27563100	1.86022800	0.00000000
Al	−0.29879400	0.65583400	−2.25114100

Al	2.23085700	−1.28798600	0.00000000
S	1.99791600	3.46049200	0.00000000
S	−1.99791600	3.46049200	0.00000000
S	−3.99583200	0.00000000	0.00000000
S	−1.99791600	−3.46049200	0.00000000
S	1.99791600	−3.46049200	0.00000000
S	3.99583200	0.00000000	0.00000000

Al	−0.29879400	0.65583400	2.25114100
S	−0.29879400	−1.19024400	−3.44999300
S	−1.71777900	2.35936800	−2.07068900
S	−1.46252100	−2.68288200	0.00000000
S	−0.29879400	−1.19024400	3.44999300
S	1.87191700	−1.95506900	0.00000000
S	−1.71777900	2.35936800	2.07068900

3C $PtAl_6S_6$ (C_1, ^1A)

Pt	0.05247200	−0.00061700	−0.63331700
Al	−1.76471900	−1.22313000	0.66700400
Al	1.19629800	2.06935000	0.13665600
Al	2.48106600	−0.24797600	−0.15145300
Al	−2.72248300	0.90815200	−1.54794700
Al	0.82720600	−2.17848300	0.27878800
Al	−1.34481300	1.74899700	0.53651900
S	3.02644300	−2.27471300	0.46485700
S	−0.79230400	−3.02329100	1.47858900
S	−0.10124500	3.44692100	1.22411300
S	−2.78426400	0.45020000	1.66276300
S	3.36448300	1.75269900	−0.07372700
S	−1.89036500	−1.22379600	−1.60382400

3D $PtAl_6S_6$ (C_1, ^1A)

Pt	0.03331900	−0.67736100	−0.48412800
Al	−2.02280500	−1.71513400	−1.23071300
Al	−0.16682700	1.69701500	−0.99546800
Al	2.30480300	0.72133100	−0.15637200
Al	−2.20650100	0.47041500	0.49944800
Al	1.89331300	−1.84924600	0.79039500
Al	0.26846200	0.05595200	1.81562300
S	2.44594600	0.42982200	2.10737300
S	−3.76482500	−1.00896700	−0.17020500
S	3.05003200	−1.17695300	−1.00205700
S	−1.62164300	0.54996100	2.68164800
S	1.81520300	2.58234800	−1.22418400
S	−2.14437800	2.42940400	−0.61981600

3E $PtAl_6S_6$ (C_s, $^1A'$)

Pt	0.81711800	0.02872600	0.00000000
Al	−1.99342800	0.15528700	0.00000000
Al	−0.65541600	0.83073000	2.08217400
Al	−0.20348100	−1.82429200	−1.39334100
Al	−0.20348100	−1.82429200	1.39334100
Al	−0.65541600	0.83073000	−2.08217400
Al	0.16631900	2.38979900	0.00000000
S	−1.97181100	−2.24538200	0.00000000
S	1.45245400	−2.48410500	0.00000000
S	−0.20348100	−0.93246900	−3.38828000
S	−0.20348100	−0.93246900	3.38828000
S	−0.08844800	3.00052200	2.08384800
S	−0.08844800	3.00052200	−2.08384800

Table S2. Orbital composition analysis for the canonical molecular orbitals (CMOs) of GM $NiAl_6S_6$ (**1**, D_{6h}, $^1A_{1g}$) cluster. Main components greater than 15% are shown in bold.

CMO	Al_6 (%)		S_6 (%)		Ni (%)	
	s/p	total	s/p	total	s/p/d	total
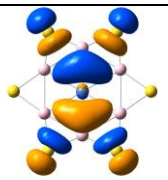 HOMO (e_{1u})	11.64/**20.38**	**32.02**	0.00/**43.27**	**43.27**	0.00/**22.85**/0.00	**22.85**
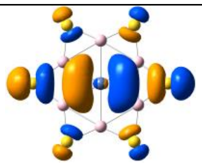 HOMO′ (e_{1u})	11.64/**20.39**	**32.03**	0.00/**43.2**9	**43.29**	0.00/**22.85**/0.00	**22.85**
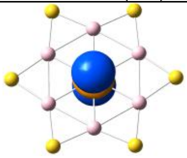 HOMO-1 (a_{1g})	0.00/3.89	3.89	0.00/0.00	0.00	0.00/0.00/**85.47**	**85.47**
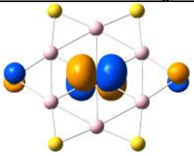 HOMO-2 (e_{1g})	0.00/0.00	0.00	0.00/9.06	9.06	0.00/0.00/**90.55**	**90.55**
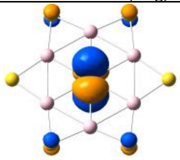 HOMO-2′ (e_{1g})	0.00/0.00	0.00	0.00/9.06	9.06	0.00/0.00/**90.55**	**90.55**
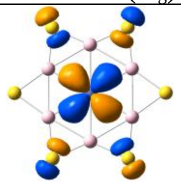 HOMO-3 (e_{2g})	0.00/0.00	0.00	0.00/**15.95**	**15.95**	0.00/0.00/**81.88**	**81.88**

CMO	Al_6 (%)		S_6 (%)		Ni (%)	
	s/p	total	s/p	total	s/p/d	total
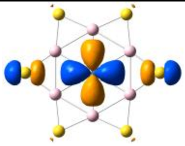 HOMO-3′ (e_{2g})	0.00/0.00	0.00	0.00/**15.87**	**15.87**	0.00/0.00/**81.88**	**81.88**
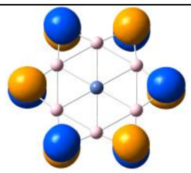 HOMO-4 (b_{1g})	0.00/0.00	0.00	0.00/**97.64**	**97.64**	0.00/0.00/0.00	0.00
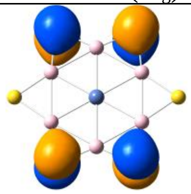 HOMO-5 (e_{2u})	0.00/7.87	7.87	0.00/90.33	**90.33**	0.00/0.00/0.00	0.00
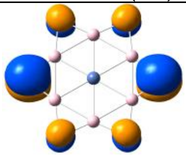 HOMO-5′ (e_{2u})	0.00/7.87	7.87	0.00/**90.33**	**90.33**	0.00/0.00/0.00	0.00
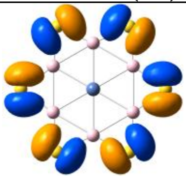 HOMO-6 (a_{2g})	0.00/6.05	6.05	0.00/**91.37**	**91.37**	0.00/0.00/0.00	0.00
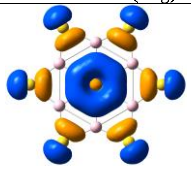 HOMO-7 (a_{1g})	0.00/14.37	14.37	0.00/**57.38**	**57.38**	14.05/0.00/10.19	**24.24**

CMO	Al_6 (%)		S_6 (%)		Ni (%)	
	s/p	total	s/p	total	s/p/d	total
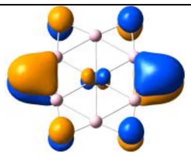 HOMO-8 (e_{1g})	0.00/**20.54**	**20.54**	0.00/**70.32**	**70.32**	0.00/0.00/7.97	7.97
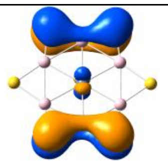 HOMO-8′ (e_{1g})	0.00/**20.54**	**20.54**	0.00/**70.32**	**70.32**	0.00/0.00/7.97	7.97
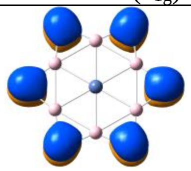 HOMO-9 (a_{2u})	0.00/**28.41**	**28.41**	0.00/**70.06**	**70.06**	0.00/0.52/0.00	0.52
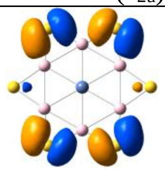 HOMO-10 (e_{1u})	2.17/10.10	12.27	0.00/**84.28**	**84.28**	0.00/0.00/0.00	0.00
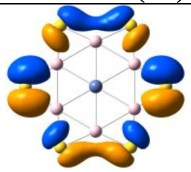 HOMO-10′ (e_{1u})	1.45/11.77	13.22	0.00/**84.25**	**84.25**	0.00/0.00/0.00	0.00
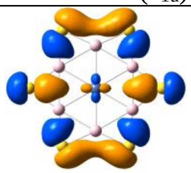 HOMO-11 (e_{2g})	1.22/**16.50**	**17.72**	1.22/**74.09**	**75.31**	0.00/0.00/4.23	4.23

CMO	Al_6 (%)		S_6 (%)		Ni (%)	
	s/p	total	s/p	total	s/p/d	total
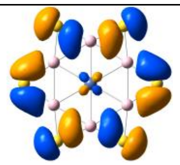 HOMO-11′ (e_{2g})	0.00/1**6.49**	1**6.49**	0.00/**74.09**	**74.09**	0.00/0.00/4.23	4.23
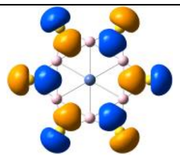 HOMO-12 (b_{2u})	0.00/9.58	9.58	7.77/**79.07**	**86.84**	0.00/0.00/0.00	0.00
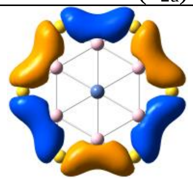 HOMO-13 (b_{1u})	**19.94**/7.53	**27.47**	0.00/**68.86**	**68.86**	0.00/0.00/0.00	0.00
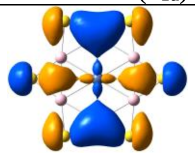 HOMO-14 (e_{2g})	**19.59**/0.00	**19.59**	8.19/**59.64**	**67.83**	0.00/0.00/6.85	6.85
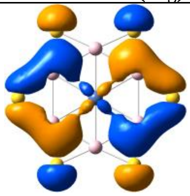 HOMO-14′ (e_{2g})	**19.59**/1.81	21.40	8.18/**59.63**	**67.81**	0.00/0.00/6.85	6.85
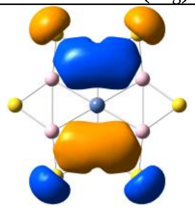 HOMO-15 (e_{1u})	**24.57**/10.03	**34.60**	13.15/**43.28**	**56.43**	0.00/5.35/0.00	5.35

CMO	Al_6 (%)		S_6 (%)		Ni (%)	
	s/p	total	s/p	total	s/p/d	total
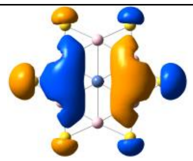 HOMO-15′ (e_{1u})	**24.58**/10.67	**35.25**	13.15/**43.15**	**56.30**	0.00/5.35/0.00	5.35
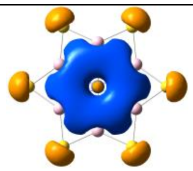 HOMO-16 (a_{1g})	**24.50**/**24.36**	**48.86**	12.13/**20.96**	**33.09**	11.24/0.00/3.52	14.76
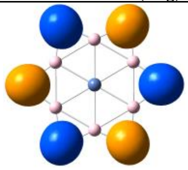 HOMO-17 (b_{2u})	0.00/**17.45**	**17.45**	**80.56**/0.00	**80.56**	0.00/0.00/0.00	0.00
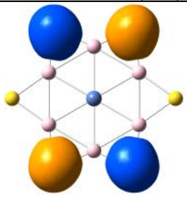 HOMO-18 (e_{2g})	3.06/**15.55**	**18.61**	**78.46**/0.00	**78.46**	0.00/0.00/0.00	0.00
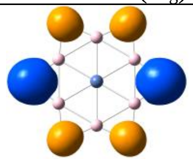 HOMO-18′ (e_{2g})	2.04/14.97	**17.01**	**77.28**/1.20	**78.48**	0.00/0.00/0.00	0.00
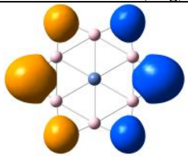 HOMO-19 (e_{1u})	12.14/7.78	**19.92**	**74.31**/1.87	**76.18**	0.00/0.00/0.00	0.00

CMO	Al_6 (%)		S_6 (%)		Ni (%)	
	s/p	total	s/p	total	s/p/d	total
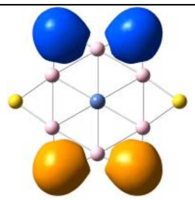 HOMO-19′ (e_{1u})	12.14/7.78	**19.92**	**75.44**/0.00	**75.44**	0.00/0.00/0.00	0.00
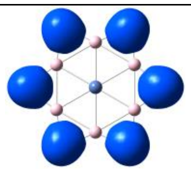 HOMO-20 (a_{1g})	**19.01**/0.00	**19.01**	**74.24**/1.13	**75.37**	0.78/0.00/0.00	0.78

Table S3. Orbital composition analysis for the lowest unoccupied molecular orbitals (LUMOs) of the MAl_6S_6 (M = Ni, Pd, Pt) series.

Species	LUMO	Symmetry	Al_6 (%)	S_6 (%)	M (%)
**1** $NiAl_6S_6$	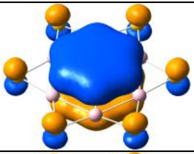	a_{2u}	52.72	25.18	22.09
**2** $PdAl_6S_6$	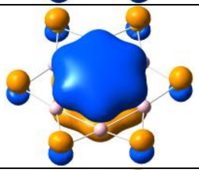	a_{2u}	43.88	23.87	32.23
**3** $PtAl_6S_6$	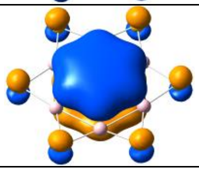	a_{2u}	42.12	23.98	33.87

Figure S1. The AdNDP bonding pattern of $PdAl_6S_6$. Occupation numbers (ONs) are shown.

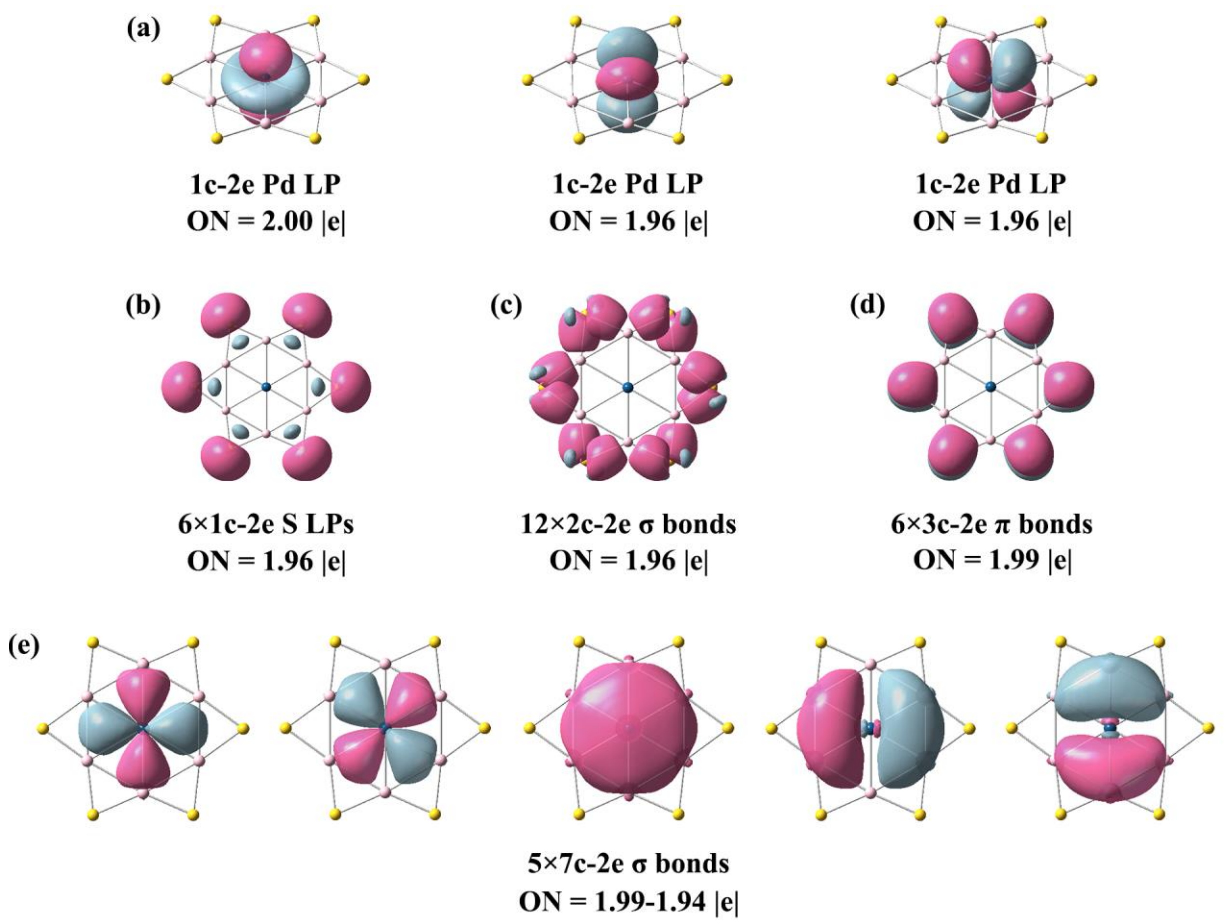

Figure S2. The AdNDP bonding pattern of $PtAl_6S_6$. Occupation numbers (ONs) are shown.

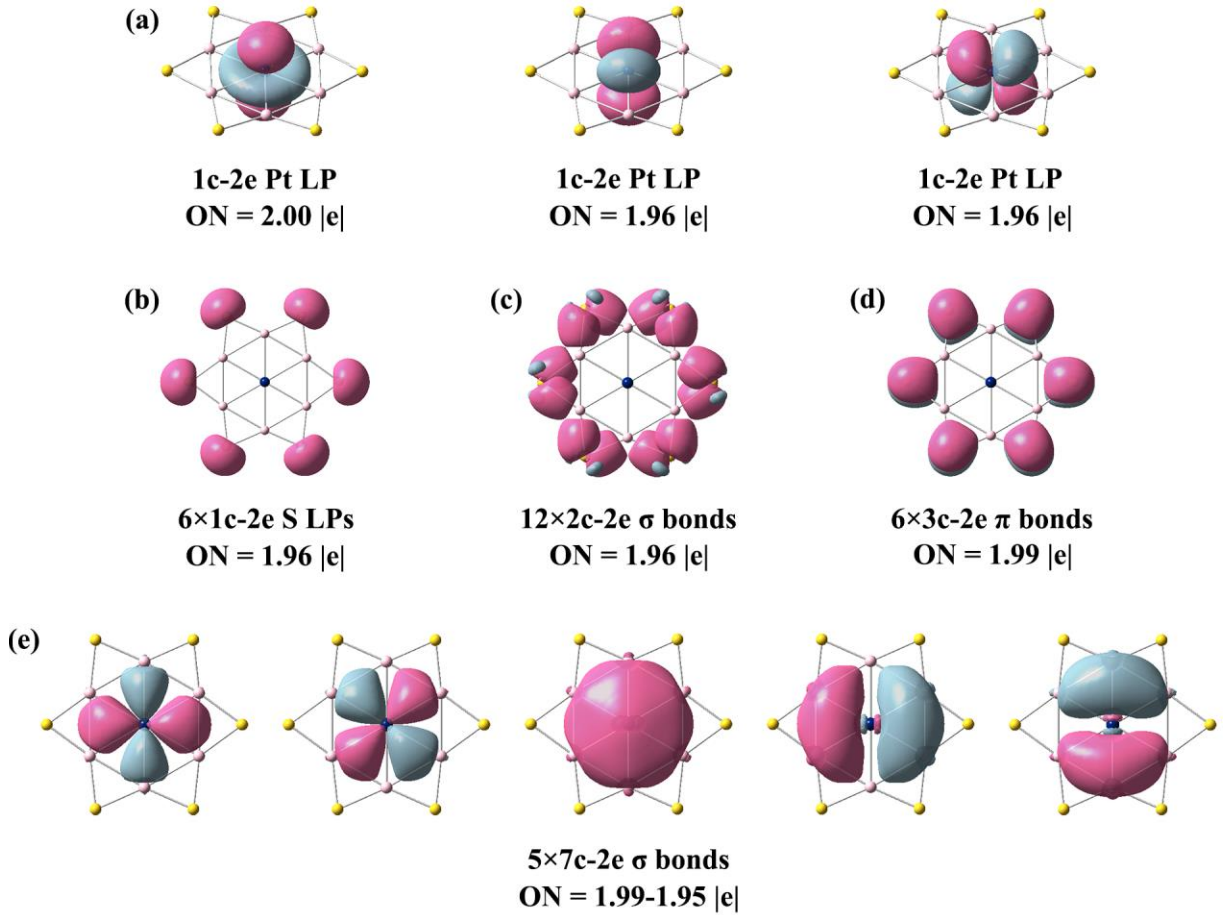

Figure S3. Nucleus independent chemical shifts (NICSs) for clusters **1**–**3**. Here the NICS(0) data (blue color) are calculated at the center of the Al-M-Al (M = Ni, Pd, Pt) triangle, whereas the NICS(1) values (red color) are at 1 Å above the center of the Al-S-Al triangle.

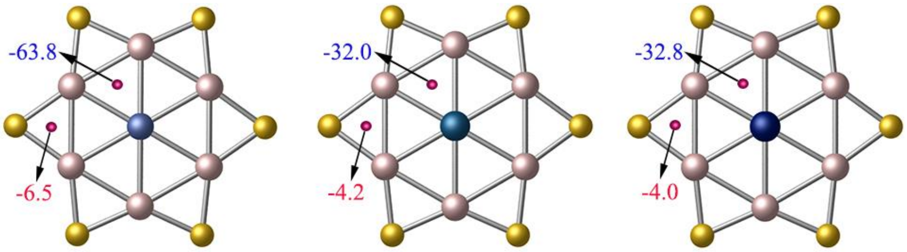

1 D_{6h} $NiAl_6S_6$ **2** D_{6h} $PdAl_6S_6$ **3** D_{6h} $PtAl_6S_6$

Figure S4. Calculated IR spectrums of $PdAl_6S_6$ and $PtAl_6S_6$ at the PBE0/def2-TZVP level.

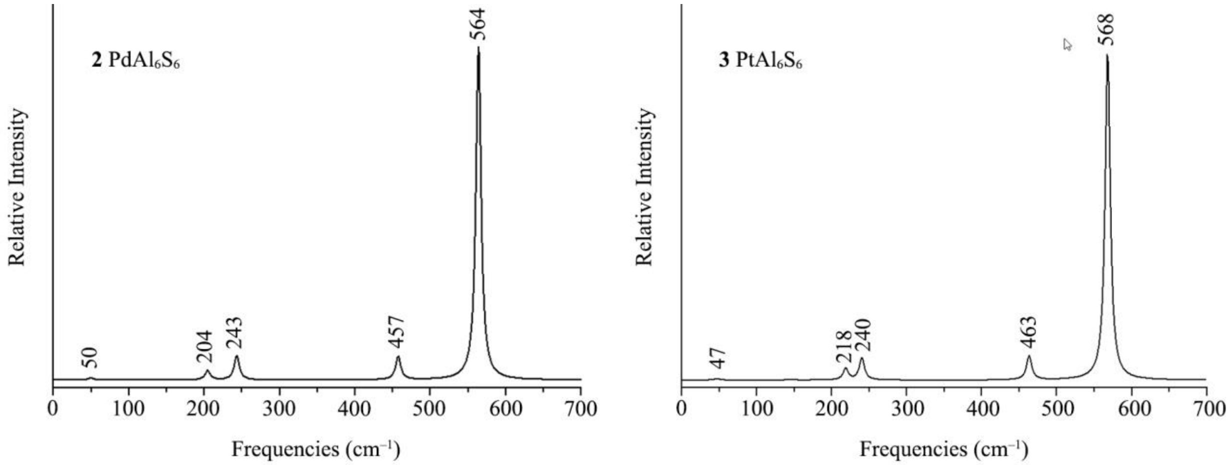

Supplement: Supplementary file 1 [file molecules-28-00942-s001.zip › molecules-2148610-supplementary.pdf]
